# p53 partial loss-of-function mutations sensitize to chemotherapy

**DOI:** 10.1038/s41388-021-02141-5

**Published:** 2021-12-14

**Authors:** Boris Klimovich, Nastasja Merle, Michelle Neumann, Sabrina Elmshäuser, Andrea Nist, Marco Mernberger, Daniel Kazdal, Albrecht Stenzinger, Oleg Timofeev, Thorsten Stiewe

**Affiliations:** 1 Institute of Molecular Oncology, Universities of Giessen and Marburg Lung Center (UGMLC), Member of the German Center for Lung Research (DZL), Philipps-University, Marburg, Germany; 2 Genomics Core Facility, Philipps-University, Marburg, Germany; 3 Institute of Pathology, Heidelberg University Hospital, Translational Lung Research Center Heidelberg (TLRC-H), Member of the German Center for Lung Research (DZL), Heidelberg, Germany

**Keywords:** Cancer models, Chemotherapy, Oncogenes

## Abstract

The tumor suppressive transcription factor p53 is frequently inactivated in cancer cells by missense mutations that cluster in the DNA binding domain. 30% hit mutational hotspot residues, resulting in a complete loss of transcriptional activity and mutant p53-driven chemotherapy resistance. Of the remaining 70% of non-hotspot mutants, many are partial loss-of-function (partial-LOF) mutants with residual transcriptional activity. The therapeutic consequences of a partial-LOF have remained largely elusive. Using a p53 mutation engineered to reduce DNA binding, we demonstrate that partial-LOF is sufficient to enhance oncogene-driven tumorigenesis in mouse models of lung and pancreatic ductal adenocarcinoma and acute myeloid leukemia. Interestingly, mouse and human tumors with partial-LOF mutations showed mutant p53 protein accumulation similar as known for hotspot mutants. Different from the chemotherapy resistance caused by p53-loss, the partial-LOF mutant sensitized to an apoptotic chemotherapy response and led to a survival benefit. Mechanistically, the pro-apoptotic transcriptional activity of mouse and human partial-LOF mutants was rescued at high mutant protein levels, suggesting that accumulation of partial-LOF mutants enables the observed apoptotic chemotherapy response. p53 non-hotspot mutants with partial-LOF, therefore, represent tumorigenic p53 mutations that need to be distinguished from other mutations because of their beneficial impact on survival in a therapy context.

## Introduction

The tumor suppressor gene *TP53* is mutated in roughly half of all cancer patients [1]. In contrast to other tumor suppressor genes, *TP53* mutations are most often missense mutations giving rise to mutant proteins which accumulate in tumor cells to high levels. The *TP53* gene product p53 responds to various types of cellular stress, DNA damage being the most prominent, and functions as a transcription factor that binds DNA in a sequence-specific manner to regulate a host of transcriptional programs, any or all of which can contribute to suppressing tumorigenesis [2]. Reflecting the functional importance of p53 DNA binding for tumor suppression, missense mutations cluster in the exons encoding the DNA binding domain (DBD). Overall, more than 2000 different missense variants have been reported in cancer cells and yield a complex mutation spectrum [1, 3].

Some codons like R175, R248 and R273 are more frequently mutated than others and the top 10 ‘hotspot’ mutations together account for ~30% of all missense mutations. The hotspot mutations are structurally well-characterized and subdivided into ‘contact’ mutations, which remove essential DNA-contact residues, and ‘structural’ mutations affecting residues that are critical for the overall architecture of the DNA binding protein surface [4]. Studies in cell culture and knock-in mouse models have yielded a thorough mechanistic understanding of the functional impact of hotspot mutations, which have largely lost the tumor suppressive activity of the wild-type proteins (LOF, loss-of-function) and, by oligomerization, exert additional dominant-negative activity towards wild-type p53 expressed from a remaining non-mutated allele [5]. Moreover, several hotspot mutants have acquired neomorphic properties (also termed gain-of-function, GOF) that, in an oncogene-like fashion, actively promote tumor progression to a more aggressive and therapy-resistant state [6–8]. These properties of mutant p53 proteins help to explain the preference for missense over null mutations and the poor prognosis associated with p53 mutations in several cancer types [9].

Importantly, our current knowledge on mutant p53 is mostly based on studies of hotspot mutants. The functional consequences of non-hotspot mutants, that comprise the majority (~70%) of all patients with *TP53* missense mutations, are still poorly understood. A landmark study that systematically profiled the transcriptional activity of 2314 p53 variants in a yeast-based reporter assay revealed substantial differences in transactivation [10]. Loss of transactivation correlated with mutant frequency supporting a selection bias for loss-of-transactivation mutants during cancer development. In other words, while hotspot mutants completely lack transcriptional activity, the less frequent non-hotspot mutants tend to retain residual activity which was independently confirmed for a set of selected mutants in yeast and mammalian cells [11, 12]. In line, more recent systematic screens based on mutant p53 cDNA expression in human tumor cell lines have observed highly heterogenous antiproliferative activity among thousands of p53 variants [13, 14]. The loss of antiproliferative activity was more pronounced for hotspot than non-hotspot mutants, confirming that non-hotspot variants are often only partial loss-of-function (partial-LOF) variants. Of note, mice carrying a hypomorphic p53 allele or the murine partial-LOF variant R172P (corresponding to human R175P) succumb prematurely to cancer proving that partial-LOF mutations are pathogenic despite their residual tumor suppressive activity [15, 16]. However, the extent of cancer susceptibility is strongly context-dependent. For example, the murine R172P mutant is apoptosis-deficient and fails to prevent tumorigenesis in a mouse model of *Myc*-driven B-cell lymphomagenesis [17], where p53 is known to limit tumorigenesis primarily by means of apoptosis [18]. In contrast, R172P efficiently suppresses *Kras*^G12D^-triggered development of pancreatic ductal adenocarcinoma (PDAC) through its residual senescence-inducing activity [19]. Together these findings suggest that partial-LOF and LOF mutants promote the development of different tumor types so that partial-LOF mutations should be more frequent in, for example, Burkitt lymphoma with chromosomal *Myc*-translocations than in *Kras*-driven PDAC. However, this hypothesis is challenged by a similar overall frequency of p53 partial-LOF mutations in different cancer types [20].

Mutations at R175 (mouse R172) affect zinc coordination in the DBD, but our structural understanding of the partial-LOF associated with R175P versus a complete LOF in R175H remains still incomplete [21]. Mechanistically better understood are non-hotspot mutations affecting the DBD surface residues E180 and R181 (mouse E177 and R178). These residues form an intermolecular salt-bridge which stabilizes the DNA-bound tetramer and enables p53 DBDs to bind response elements in a cooperative manner [20, 22–26]. Several mutations of these residues have been described as somatic or germline variants in ∼0.5% of tumors with frequencies of individual variants up to 0.114% in the case of R181C [1, 3, 20]. Based on the world-wide cancer incidence, all E180/R181 mutations together account for an estimated number of 34,000 cancer patients per year [20]. The distribution of these cooperativity mutations across different tumor types is similar as for hotspot mutations and other frequent p53 mutations supporting a comparable causal role as drivers of tumorigenesis [20]. The charge-inverting E180R mutation is not a single-nucleotide variant and has therefore not been found in cancer patients so far, but it is mechanistically characterized better than any other cooperativity mutant regarding protein structure, cooperative DNA binding, and target gene activation [23, 27, 28]. In addition, the murine equivalent E177R is available as a knock-in mouse for in vivo studies [29, 30]. E177R mice show a preferential defect in apoptosis induction resulting in increased susceptibility to sporadic and Myc-driven lymphomagenesis [30, 31]. Moreover, the E177R mutant phenotype is explained entirely by a lack of DNA binding cooperativity as it is fully rescued in vitro and in vivo by complementation with the human R181E (mouse R178E) mutant [23, 27, 28, 32].

Here we have used the E177R mutant knock-in mouse along with a panel of cancer patient-derived cooperativity mutants to explore the role of a partial LOF in tumorigenesis and cancer therapy. We demonstrate that a p53 partial-LOF cooperates with oncogenes to drive tumorigenesis in multiple tissues. Most interestingly, we found that the residual transcriptional activity of partial-LOF mutants is retained in tumors and can be therapeutically boosted to provide a beneficial therapy outcome, superior to LOF mutations.

## Results

### Non-hotspot mutations are enriched for partial loss-of-function variants

**Fig. 1:**
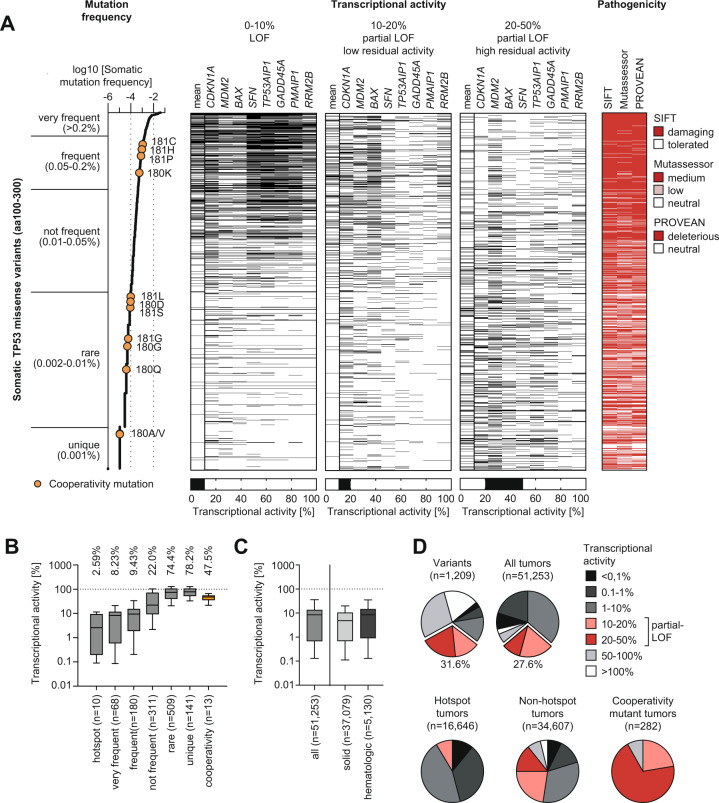
Prevalence of p53 partial-LOF mutations in human cancer patients. **A** Frequency, transcriptional activity, and in silico predicted pathogenicity of 1,209 somatic *TP53* missense mutations affecting the p53 DNA binding domain (amino acids 100–300). Three black-white heatmaps depict the mean and response element-specific transcriptional activity of missense variants. The left heatmap illustrates the distribution of loss-of-function (LOF) events (0-10% transcriptional activity), the middle heatmap partial LOF events with low (10–20%) residual activity and the right heatmap partial LOF events with high (20-50%) residual activity. The pathogenicity plot shows variants predicted to be damaging by SIFT, Mutassessor or PROVEAN in red. All data were extracted from the UMD *TP53* mutation database (http://p53.fr/tp53-database). Transcriptional activity data were determined in a yeast-based reporter assay [10]. Cooperativity mutations at residues E180 and R181 are highlighted in orange. **B** Non-hotspot *TP53* missense mutants retain substantial transcriptional activity. Shown is the range of transcriptional activity for somatic *TP53* mutations grouped according to mutation frequency in cancer patients. Cooperativity mutations are highlighted separately in orange. **C**, **D** Range and distribution of transcriptional activity in p53 mutant cancer patients. In all box plots, boxes indicate median and interquartile range, whiskers the 10–90 percentile. Pie charts depict the percentage of variants (or tumors) falling into different categories of transcriptional activity as indicated. 31.6% of p53 variants and 27.6% of tumors with p53 missense mutations fall into the partial-LOF category defined by a residual transcriptional activity of 10–50%.

We extracted from the UMD *TP53* mutation database (https://p53.fr/tp53-database) a total of 1209 *TP53* missense mutations, that were identified in patient tumor samples at least once and map to the DNA binding domain (aa100-300), along with their transcriptional activity as measured in a yeast-based reporter assay using response elements (REs) of 8 prototypical p53 target genes [10]. We defined a loss of transcriptional activity (loss-of-function, LOF) as less than 10% residual activity and partial loss-of-function (partial-LOF) with low (10–20%) or high (20-50%) residual activity. When plotting the transcriptional activity of mutants sorted by their frequency in the set of tumor samples, we noted the characteristic enrichment of LOF variants in the mutants with a high frequency of >0.05% (Fig. 1A and B). Importantly, we also observed an unexpectedly high number of partial-LOF variants with low or high residual transcriptional activity in the various mutant frequency groups (Fig. 1A and B). In particular, mutants with medium abundance in cancer patients often display partial-LOF. While the median transcriptional activity of hotspots mutants was strongly reduced to 2.59% of the wild-type, the other frequency classes show substantially higher median residual activity ranging between 8.23% for very frequent mutants and 78.2% for unique variants (Fig. 1B). This was observed for the calculated median transcriptional activity (Fig. 1B), but also for the transcriptional activity at all individual tested REs (Supplementary Fig. S1A). The same trend is observed when analyzing the relative fitness score (RFS) of variants which reflects their loss of antiproliferative activity and was measured upon enforced expression in p53-null H1299 cells [13]. Again, many of the mutants with medium abundance in the cancer population often show an intermediate RFS (−2<RFS < 0) indicative of residual antiproliferative activity (Supplementary Fig. S1B). Supporting the idea that partial-LOF mutants are driver mutations rather than neutral bystanders or sequencing artefacts, mutations in all the different frequency groups are marked as damaging in the UMD p53 mutation database based on several pathogenicity prediction algorithms such as SIFT, MutAssessor and PROVEAN (Fig. 1A, right panel).

When assessing transcriptional activity on the basis of individual patient tumors – not p53 variants – the distribution is similar, revealing a large proportion of *TP53* mutant malignancies (both solid and hematological) with an intermediate p53 mutant transcriptional activity (Fig. 1C and D; Supplementary Fig. S1C and D). A total of 31.6% of variants and 27.6% of tumors with *TP53* missense mutations fall within the partial-LOF activity range (Fig. 1D). When excluding the top 10 hotspot mutants, the percentage of non-hotspot tumors harboring a partial-LOF mutant increases to 36.8% (Fig. 1D). In summary, p53 mutants with residual transcriptional activity, i.e., partial-LOF, are common in p53-mutated tumors and particularly prevalent among non-hotspot mutants.

### p53 cooperativity mutations as a model for partial-LOF mutations

**Fig. 2:**
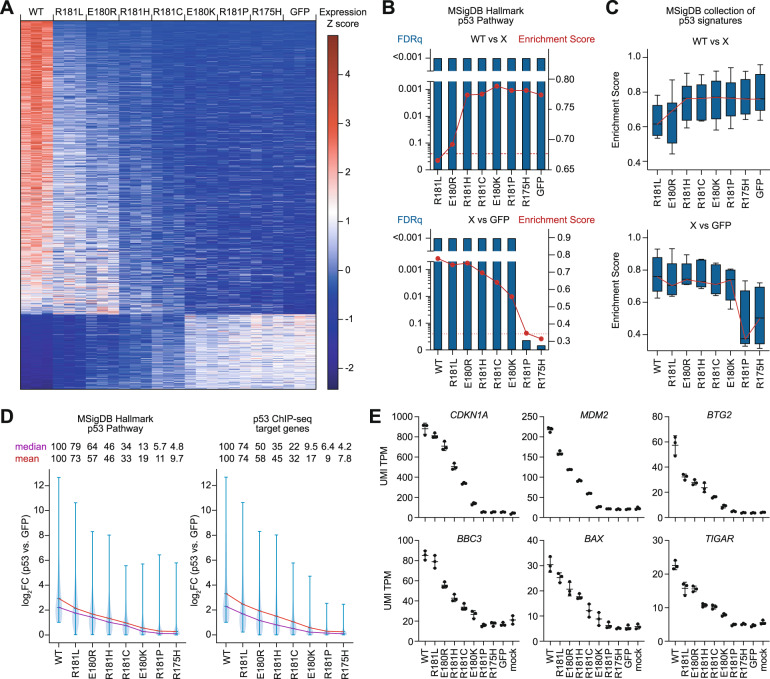
Partial loss of transcriptional activity in p53 cooperativity mutations. **A** Heat-map shows z-transformed RNA expression values (FPKM) for 1592 differentially expressed genes (mean log_2_FC ≥ 1, corrected p-value ≤ 0.05) detected by RNAseq in p53-null Saos-2 cells transduced with wild-type p53, indicated p53 missense mutants or GFP as negative control. *n* = 3 for each genotype. **B** RNAseq data from **A** were used for gene set enrichment analysis (GSEA). Depicted are the false discovery rate (FDRq, blue bars) and enrichment score (red curve) for the MSigDB Hallmark p53 Pathway gene set in pairwise comparisons between cells expressing wild-type p53 and different mutants (X) (upper panel) or all variants (X) vs. GFP-transfected (p53-null) cells (lower panel). **C** Depicted is the distribution of enrichment scores of multiple p53-related signatures of the MSigDB collection (Table S1) in pairwise comparisons like in **B**. **D** Expression of p53-target genes in WT- or mutant p53-transfected cells relative to GFP-transfected (p53-null) cells. Violin plots show the distribution of log_2_FC values for genes belonging to the MSigDB Hallmark p53 Pathway gene set (left panel) and a gene set obtained from p53 ChIP-seq (right panel, Table S1). Red and purple lines indicate mean and median log_2_FC, respectively. **E** Expression of prototypical p53 target genes obtained from **A**. UMI TPM, unique molecular identifier tags per million. Shown is the mean±SD of *n* = 3 biological replicates.

To better explore the in vivo consequences of *TP53* partial-LOF mutations, we focused on the mechanistically well-studied subclass of cooperativity mutations (labelled orange in Fig. 1A). They commonly reduce DNA binding and show a median transcriptional activity of 47.5% (13.7-67.4%) in the yeast-based reporter assay (Figs. 1B and D), classifying them as non-hotspot mutants with partial-LOF. Given that yeast are lacking p53-relevant transcriptional components and properties present in humans, we aimed to further characterize the transcriptional activity of cooperativity mutants in a more physiological system. For this, we expressed the cancer patient-derived mutants E180K, R181L, R181H, R181C, R181P, and the engineered mutant E180R in comparison to WT, the R175H hotspot mutant, and GFP as a negative control in p53-deficient Saos-2 osteosarcoma cells. Transcriptome analysis by RNA-seq revealed more genes to be activated by WT than repressed (Fig. 2A), in line with p53 being primarily a transactivator [33]. Compared to WT, all cooperativity mutants showed a strongly reduced regulation of target genes, but individual mutants differed substantially forming a continuum of residual activity (Fig. 2A). R181L, E180R, and R181H showed the highest residual activity, whereas R181P – consistent with proline functioning as a helix breaker – displayed a complete LOF indistinguishable from R175H and GFP (Fig. 2A-E). Consistent with distinct degrees of LOF, functional annotation analysis revealed a varying enrichment of p53-related expression signatures such as the Hallmark p53 pathway gene set from the Molecular Signatures Database (MSigDB) in WT transcriptomes when compared to each one of the mutants (Fig. 2B and C, Supplementary Table 1). Importantly, p53-related gene sets were also significantly enriched in R181L, E180R, R181H, R181C, and E180K mutant transcriptomes compared to the GFP control, highlighting their residual transcriptional activity, i.e. their partial-LOF phenotype (Fig. 2B and C). In detail, partial-LOF cooperativity mutants displayed 5–79% residual transactivation of genes belonging to the Hallmark p53 pathway set or genes that were found to be directly bound and regulated by WT in Saos-2 cells (Fig. 2D). These findings were confirmed for the prototypical p53 target genes *CDKN1A* (p21), *MDM2*, *BTG2*, *BBC3* (Puma), *BAX,* and *TIGAR* (Fig. 2E).

Of note, the ability of cooperativity mutants to repress genes mirrored their transactivation potential (Fig. 2A). Gene set enrichment analysis indicated these repressed genes to be significantly enriched for E2F and Myc target genes sets related to cell proliferation (Supplementary Fig. 2, Supplementary Table 1). Similar as seen above for transactivated gene sets, these repressed gene sets were enriched in the WT transcriptome compared to most of the mutants and enriched in R181L, E180R, R181H, R181C, and E180K compared to the GFP control attesting to their partial-LOF with respect to gene repression. Although p53 can function as a direct transrepressor [34], the majority of cell proliferation genes are known to be repressed indirectly through p53-mediated transactivation of the p21-DREAM pathway [33]. The impaired target gene repression by partial-LOF mutants is therefore most likely a consequence of their reduced transactivation potential.

In all these analyses, the E180R mutant integrated into the continuum of transcriptional activities displaying a level of residual activity in between the cancer-derived mutants R181L and R181H, which validates the E180R (mouse E177R) cooperativity mutant as a suitable model for non-hotspot mutants with partial-LOF.

### p53 partial loss-of-function cooperates with Ras oncogenes in tumorigenesis

**Fig. 3:**
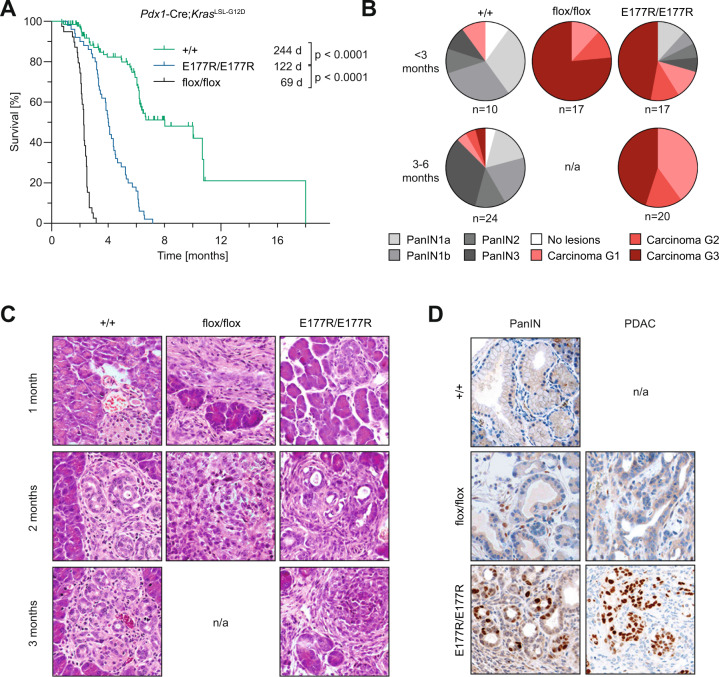
Trp53E177R mutant in Ras-driven PDAC. **A** Kaplan-Meier curves show overall survival of mice with indicated p53 genotypes and pancreas-specific expression of oncogenic mutant *Kras*^G12D^. *Trp53*^+/+^ *n* = 45, *Trp53*^E177R/E177R^ *n* = 50, *Trp53*^flox/flox^ *n* = 39, log-rank Mantel-Cox test. **B** Pie charts show percentage of mice with (pre)neoplastic pancreas lesions (highest grade that was detected in each sample). Samples were collected at different time-points from animals as in **A**. PanIN, pancreatic intraepithelial neoplasia. n/a, no sample available for analysis as all *Trp53*^flox/flox^ mice were dead by 3 months of age. **C** Representative micrographs of pancreas samples from **B**, hematoxylin and eosin (H&E) staining. **D** Immunochemical staining of p53 in representative PanIN (left) and PDAC samples (right).

Previous work has demonstrated an increased susceptibility of mice with the R172P and E177R partial-LOF mutations to both sporadic and Myc-driven lymphoma [17, 30]. In light of reports that R172P-triggered senescence prevents *Kras*^G12D^-induced pancreatic ductal adenocarcinoma (PDAC) [19], we also analyzed the E177R cooperativity mutant in this model. We noted that E177R extended the median survival from 69 days in p53^flox/flox^ mice to 122 days indicative of tumor-suppressive activity. However, p53^+/+^ animals survived twice as long (Fig. 3A). All E177R mice succumbed to PDAC within half a year and almost half showed high-grade PDAC by less than 3 months of age when the majority of p53^+/+^ only showed high-grade PanIN lesions which only rarely progressed to PDAC later on (Fig. 3B and C). Similar to R172H-mutant PDAC [19], both PanIN and PDAC lesions in E177R mice showed strong nuclear p53 staining, indicating that PDAC formation is not necessarily driven by the loss of p53 and is, in fact, compatible with sustained high-level expression of E177R (Fig. 3D). We conclude that E177R – different from R172P – delays, but fails to completely suppress PDAC development.

**Fig. 4:**
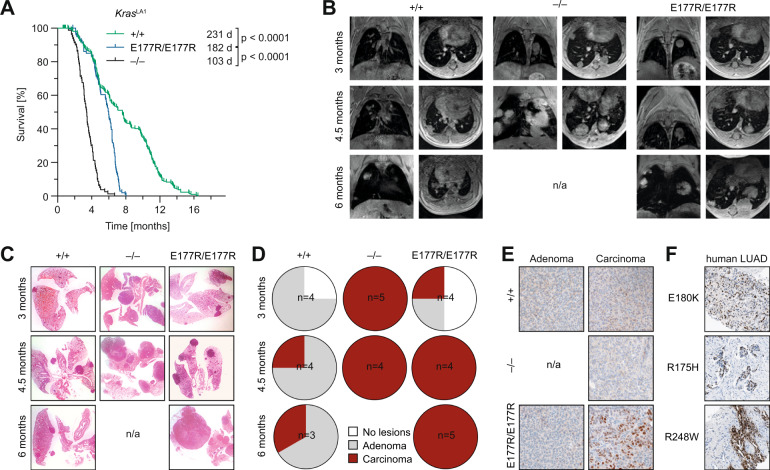
Trp53E177R mutant in Ras-driven lung cancer. **A** Kaplan-Meier curves show the overall survival of *Kras*^LA1^ mice with indicated p53 genotypes. *Trp53*^+/+^ *n* = 228, *Trp53*^E177R/E177R^ *n* = 105, *Trp53*^–/–^ *n* = 80, log-rank Mantel-Cox test. **B** A group of mice from **A** was monitored for lung tumorigenesis by MRI tomography. Representative MRI images collected at indicated time-points are shown. n/a, no mice available for analysis, as all *Trp53*^–/–^ mice were dead by 6 months of age. **C**–**E** Lung samples were collected at different time-points from *Kras*^LA1^ mice with indicated p53 genotypes. **C** Representative micrographs, H&E staining. **D** Pie charts show percentage of mice with tumors (highest grade). **E** Immunochemical staining of p53 in representative lung adenoma (left panel) and carcinoma samples (right). **F** IHC detection of mutant p53 protein in human lung adenocarcinomas (LUAD) with indicated hotspot (R175H, R248W) and non-hotspot (E180K) *TP53* mutations.

To study tumor suppression by E177R in a different Kras-driven tumor type, we extended our studies with E177R mice and crossed them to *Kras*^LA1^ mice which develop lung tumors due to spontaneous activation of a latent oncogenic *Kras*^G12D^ allele [35]. In the presence of wild-type p53 these mice develop multiple adenomas that only rarely progress to carcinomas, whereas hetero- or homoallelic inactivation of the *Trp53* gene leads to the early development of adenocarcinomas with 100% penetrance [35]. When comparing cohorts of *Kras*^LA1^ mice with different p53 genotype, we observed a significantly reduced lifespan in E177R compared to p53^+/+^ mice (Fig. 4A). Similar as observed in the PDAC model, survival of E177R mice was significantly longer in comparison to p53-null animals, indicating that residual tumor suppression by E177R slowed down *Kras*-driven tumorigenesis (Fig. 4A).

Of note, many p53-null mice with lung tumors were also burdened with thymic lymphoma, the most common tumor arising spontaneously upon loss of p53 [36]. To exclude a confounding effect on survival, we monitored lung tumor growth longitudinally with magnetic resonance imaging and analyzed lung tissues histologically at different time points (Fig. 4B–D). We observed early appearance and fast progression of adenocarcinomas in p53-deficient *Kras*^LA1^ animals, none of which reached the last time point because of the high tumor burden. In contrast, in p53 wild-type mice lung tumors were barely detectable at 3 months of age and had advanced only slowly at later time points mostly retaining a benign adenoma morphology. MRI showed presence of tumors in 2 of 4 E177R mice at 3 months, and in all animals at 4.5 months, but tumor progression was slower than in p53 knockouts. Histologically, E177R mice showed an intermediate morphology with examples of both adenoma and carcinoma at 3 months that progressed to mostly adenocarcinomas at later time points. We conclude that the residual activity of E177R provided a temporary defense against oncogenic *Kras* early during tumorigenesis, but its tumor-suppressive potential was insufficient to block progression to more malignant tumor stages.

Similar as in PDAC samples, immunohistochemical analysis showed accumulation of the E177R protein in advanced adenocarcinomas, but not in adenomas (Fig. 4E). Of note, a human lung adenocarcinoma with the E180K mutation showed strongly positive p53 immunostaining indistinguishable from hotspot mutants, confirming aberrant stabilization of a partial-LOF mutant also for human cancer tissues (Fig. 4F).

### p53 partial loss-of-function cooperates with Ras in leukemogenesis

**Fig. 5:**
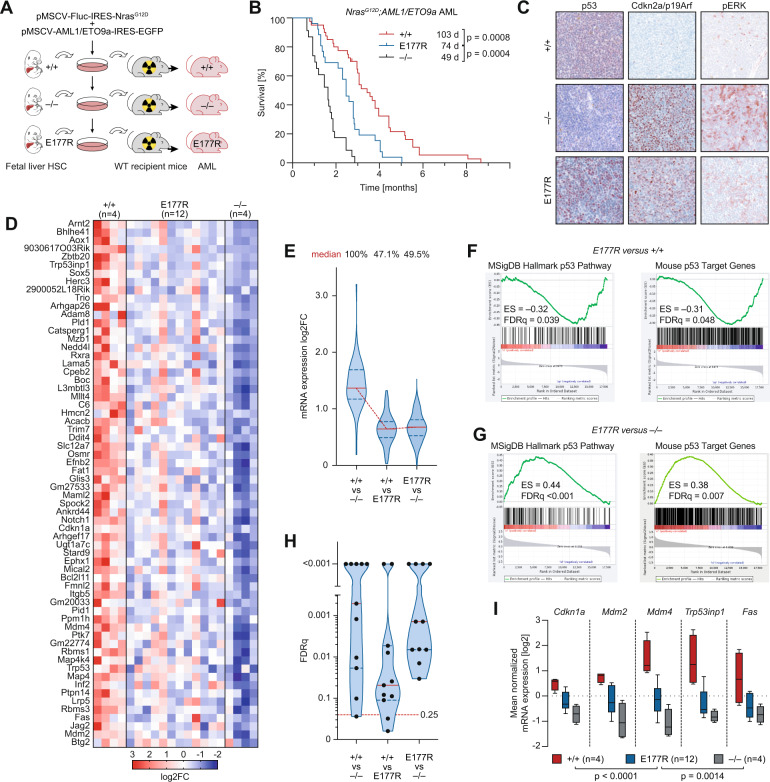
Trp53E177R mutant in leukemia mouse model. **A** Fetal liver cells were isolated from *Trp53*^+/+^, *Trp53*^E177R/E177R^, and *Trp53*^–/–^embryos at E14-16 and infected with retroviruses carrying *AML1/ETO9a* and *Nras*^*G12D*^ oncogenes co-expressed with GFP and firefly luciferase, correspondingly. After four rounds of infection, 1 million cells were transplanted into lethally irradiated (7 Gy) 129×1/SvJ albino primary recipients. **B** Kaplan–Meier survival plots for animals from **A**. *Trp53*^+/+^ *n* = 38, *Trp53*^E177R/E177R^ *n* = 26 and *Trp53*^–/–^ *n* = 23, log-rank Mantel-Cox test. **C** Representative micrographs show immunochemical staining of p53 (left panel), Cdkn2a/p19ARF (middle panel), and phospho-ERK (right panel) in spleen samples of mice with advanced leukemia. **D**, RNAseq was performed with purified AML cells isolated from terminally ill primary recipients. Shown are 62 p53 target genes defined by the presence of a p53 ChIPseq peak in wild-type MEFs and differential expression (mean log_2_FC ≥ 1) between *Trp53*^+/+^ and *Trp53*^–/–^ AML cells. **E** Violin plots (with median and interquartile range) depict expression changes between the indicated samples for all genes from **D**. **F**–**G** RNAseq data were used for gene set enrichment analysis (GSEA). Shown are enrichment plots (with enrichment scores and FDRq-values) for the denoted gene sets in pairwise comparisons of the indicated p53 genotypes. **H** Violin plots for FDRq-values from GSEA enrichment analyses show significant enrichment (FDRq < 0.25) of multiple p53-target gene sets (Table S1) in the indicated pairwise comparisons. **I** Expression of prototypical p53 target genes obtained from **A**. Box plots (Tukey) show the mean normalized mRNA expression.

Having demonstrated efficient cooperation of the E177R partial-LOF mutation with oncogenic Ras in two solid tumor models, we next explored this cooperation in a model of acute myeloid leukemia (AML), which is driven by the combination of *Nras*^*G12D*^ and *AML1/ETO9a* oncogenes and in which p53 is known to be tumor suppressive [37]. As hematopoietic bone marrow cells are in general more vulnerable to p53 activity than other cells [38–41], we speculated that E177R might be more tumor suppressive in leukemia than in solid tumors. We isolated hematopoietic fetal liver stem cells from p53^+/+^, p53^–/–^ and homozygous E177R embryos and transduced cells with two bicistronic retroviral constructs – one expressing *Nras*^*G12D*^ and firefly luciferase, the second *AML1/ETO9a* and EGFP, which allowed disease monitoring (Fig. 5A). Five independent batches of transduced hematopoietic cells of each genotype were transplanted into 5-8 lethally irradiated recipients. Consistent with published data [37], loss of p53 dramatically accelerated AML development (median survival 49 vs. 103 days, *P* < 0.0001). Mice transplanted with oncogene-transduced E177R fetal liver cells demonstrated an intermediate survival of 74 days which differed significantly from both p53^+/+^ and p53^–/–^ AML (*P* = 0.0008 and *P* = 0.0004, respectively) (Fig. 5B). As p53 is induced by oncogenes via Cdkn2a/p19ARF [42], we analyzed p19ARF in established AML samples. Immunohistochemistry revealed low levels of Cdkn2a/p19ARF and p53 in wild-type AML, indicating strong selection pressure against an intact p19ARF-p53 axis. In contrast, the E177R AML samples were positive for p19ARF like p53^–/–^ AML and accumulated high levels of mutant p53 protein (Fig. 5C). Thus, even though E177R exhibits sufficient residual activity to significantly delay leukemogenesis, the developing leukemia cells eventually tolerate E177R expression and do not experience selective pressure to uncouple it from activating oncogenic signals transmitted through p19ARF.

Global transcriptome profiling of primary leukemia samples by RNA-seq revealed an intermediate level of transcriptional p53 activity in E177R mutant AML (Fig. 5D). When examining a set of direct p53 target genes that contain validated p53 binding sites [32] and that are differentially expressed between p53^+/+^ and p53^–/–^ AML, their median expression is reduced in E177R AML by 47.1% (Fig. 5D and E). Indicative of a loss of function, canonical p53 signatures were significantly enriched in p53^+/+^ compared with E177R AML (Fig. 5F–H). Nevertheless, confirming residual transcriptional activity of E177R, the same signatures were significantly enriched in E177R vs. p53^–/–^ AML (Fig. 5G, H). Last but not least, several prototypical p53 target genes were expressed in E177R AML at a level in-between p53^+/+^ and p53^–/–^ AML (Fig. 5I).

We conclude that the E177R partial-LOF mutation delays, but does not prevent development of Ras-driven cancer in several mouse models. Furthermore, transcriptomic profiling of tumor samples confirms retention of residual p53 activity in such tumors along with accumulation of the mutant protein.

### p53 partial-LOF supports chemotherapy response and improves survival

**Fig. 6:**
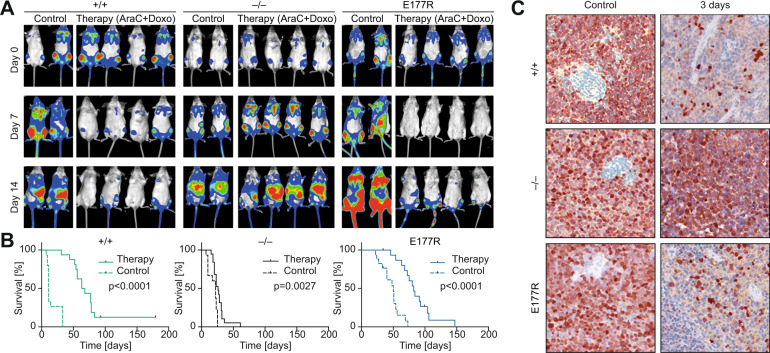
Trp53E177R mutant supports chemotherapy response and improves survival in AML. **A** Exemplary bioluminescence imaging (BLI) pictures of representative albino mice transplanted with leukemia cells of the indicated p53 genotype at indicated time points after start of chemotherapy. **B** Kaplan–Meier survival plots for control and therapy groups. *Trp53*^+/+^ control: *n* = 15, therapy: *n* = 16; *Trp53*^–/–^ control: *n* = 15, therapy: *n* = 19; *Trp53*^E177R/E177R^ control: *n* = 17, therapy: *n* = 20; *n* = 3 independent leukemias per genotype. **C** Immunohistochemical staining of GFP used as a surrogate marker of leukemia cells in spleens collected from control (left panel) and treated animals (right panel).

Despite the abundance of p53 partial-LOF mutations in cancer patients, little is known about their therapeutic implications. While p53-loss and, in particular, p53 hotspot mutants are generally considered a marker of poor therapy response and drug resistance [7, 9, 43], it is unclear if this also applies to non-hotspot mutants with residual transcriptional activity. To investigate residual tumor-suppressive functions of E177R in the context of chemotherapy, we generated cohorts of mice transplanted with p53^+/+^, p53^–/–^, and E177R AML and subjected them to a standard chemotherapy for leukemia. As expected, the therapy had only a marginal effect on p53-null leukemias that continued to progress under treatment as indicated by BLI, whereas p53^+/+^ leukemias demonstrated a very good response (Fig. 6A). Surprisingly, in all treated mice from the E177R cohort we observed a strong reduction in luciferase signal after therapy (Fig. 6A). Predictably, the wild-type p53 group showed the best therapy outcome with a median survival benefit of 54 days (*P* < 0.0001), compared to a very modest 7-day survival advantage in the p53-null cohort (*P* = 0.0405). Importantly, treated mice with E177R leukemia demonstrated a substantial survival benefit of 32 days (*P* < 0.0001, Fig. 6B). Consistently, infiltration by GFP-positive AML cells was decreased within 3 days in both p53^+/+^ and E177R spleens, confirming that E177R was not only acting in a cytostatic manner by cell cycle inhibition, but was also effectively eliminating leukemia cells (Fig. 6C).

**Fig. 7:**
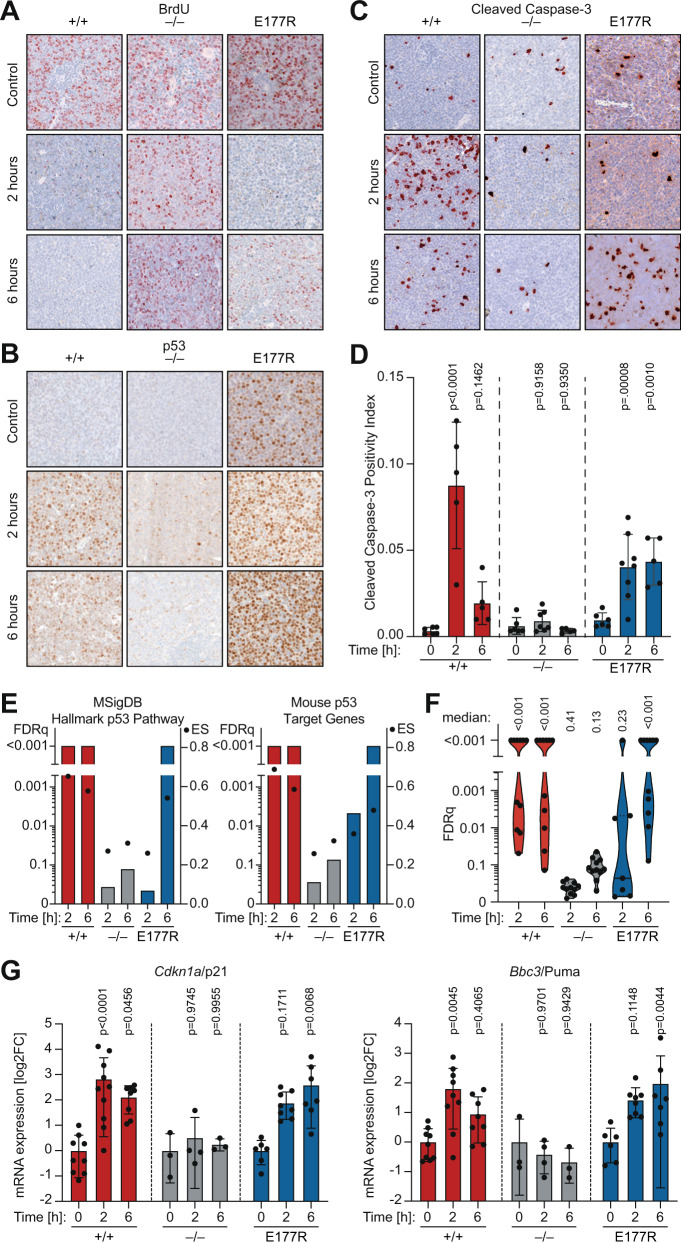
Apoptotic chemotherapy response in Trp53E177R AML. **A**–**C**, Representative IHC images of spleen samples stained for **A** BrdU as proliferation marker, **B** p53 protein, and **C** cleaved caspase 3 (CC3) as apoptosis marker. **D** Quantification of CC3 in 10 fields of view per mouse sample. Shown are mean ± SD; datapoints represent individual mice; 2way ANOVA with Dunnett’s multiple comparisons test. **E**, **F** Leukemia samples were collected from control and treated mice at 2 and 6 hours after therapy and analyzed by RNAseq and GSEA. **E** Graphs depict GSEA results for the indicated gene sets in pairwise comparisons between control and treated leukemias. Bars, false discovery rate (FDRq); dots, enrichment score. **F** Violin plots illustrate distribution of FDRq values from GSEA with multiple p53-related gene sets (Table S1). Each data point represents one gene set. **G** mRNA expression (RT–qPCR) of p53 target genes *Cdkn1a* and *Bbc3* normalized to *Actb* (β-actin). Shown is the mean ± SD log2-fold change in treated leukemia samples relative to untreated; 2way ANOVA with Dunnett’s multiple comparisons test; datapoints represent individual mice.

To explore the underlying mechanism, we collected spleen samples from control and treated mice at 2 and 6 h after start of therapy. All untreated control samples independently of genotype showed high levels of proliferation and low background levels of apoptosis (Fig. 7A–C). Upon chemotherapy p53 became rapidly stabilized in p53^+/+^ leukemias with an expression peak at 2 h (Fig. 7B). E177R expression was already high before treatment but increased further at 6 hours. Two hours after start of therapy, a strong decrease in BrdU-positive cells was detected in both p53^+/+^ and E177R leukemias, attesting to the known ability of E177R in mounting a cell cycle arrest [30], whereas p53-null leukemia retained high levels of proliferation (Fig. 7A). Moreover, we observed a massive peak in apoptosis after two hours in p53^+/+^ AML, while apoptosis levels remained low in p53-null leukemia (Fig. 7C and D). Remarkably, treated E177R AML cells also showed significant activation of apoptosis which was only slightly delayed compared to p53^+/+^ cells (Fig. 7C and D).

To gain deeper molecular insight, we analyzed untreated and treated AML samples by RNA-seq. Various p53-related transcriptional signatures, including the MSigDB Hallmark p53 Pathway, were upregulated in treated mice with high statistical significance in both p53^+/+^ and E177R, but not in p53-null leukemias (Fig. 7E and F). Similar as observed by immunohistochemistry of spleen samples, 2 h after treatment upregulation of the signatures was already highly significant in p53^+/+^ leukemias, but still variable in E177R leukemias. At 6 h, the various signatures were also uniformly enriched in E177R samples. The delayed but significant upregulation of p53 target gene expression was also confirmed by quantitative RT-PCR for the canonical target genes *Cdkn1a/p21* and *Bbc3/Puma* using a larger set of mouse samples (Fig. 7G).

### Elevated protein levels rescue the transcriptional apoptosis defect of partial-LOF mutants

**Fig. 8:**
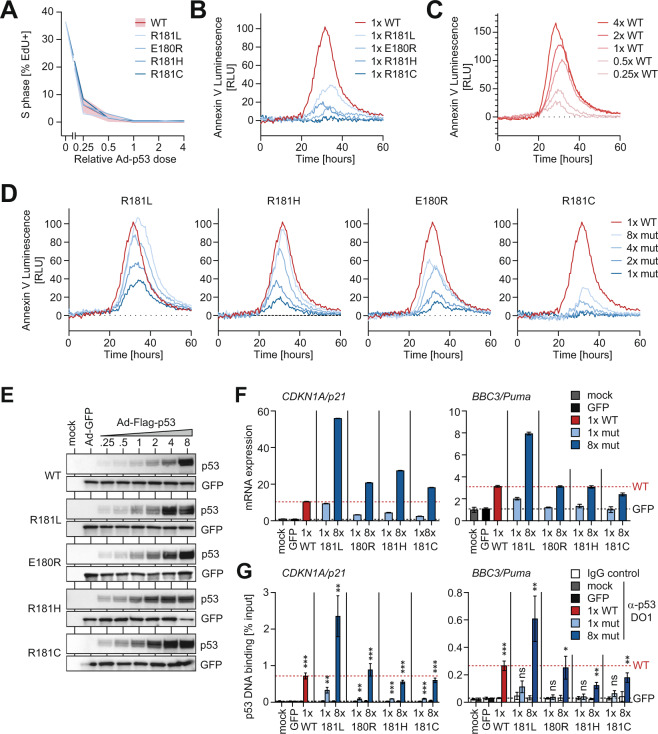
Elevated protein levels rescue the transcriptional apoptosis defect of partial-LOF mutants. Saos-2 cells were transduced with different relative amounts of p53/GFP-coexpressing vectors. Total amount of vector was adjusted to equal levels with GFP-only vector. **A** Immunofluorescence quantification of S-phase as mean percentage of EdU+ cells (*n* = 3). **B**–**D** Real-time quantification of apoptosis shown as mean Annexin V luminescence relative to GFP-only control (*n* = 3). **E** WB for p53-mutant protein levels. Equal vector load is confirmed by GFP. **F** mRNA expression of p53 target genes *CDKN1A/p21* and *BBC3/Puma* (relative to GFP-only control vector). Expression values were normalized to *GAPDH* and are shown as mean ± SD (*n* = 3 replicates). **G**, Chromatin immunoprecipitation analysis of indicated p53 variants at the *CDKN1A/p21* and *BBC3/Puma* promoter 18 hours after transduction. Chromatin samples were immunoprecipitated with α-p53 antibody or IgG as background control. Shown is DNA binding as % input chromatin (mean ± SD, *n* = 3 replicates). *P*-values (***P < 0.001; **P < 0.01; *P < 0.05; ns not significant; two-sided t-test) indicate statistical significance of the DNA binding signal relative to the respective IgG background control.

The apoptotic chemotherapy response and transcriptional induction of pro-apoptotic p53 target genes seen in treated E177R leukemia cells were rather unexpected, given that various partial-LOF mutants, including E177R, were previously described to have a selective apoptosis defect [16, 28, 30, 44–46]. However, the extent of apoptosis induced by wild-type p53 is strongly determined by the amount and dynamics of p53 protein accumulation [47–50]. We, therefore, speculated that the apoptosis deficiency of partial-LOF mutants is rescued when mutant proteins are expressed at elevated levels or with sustained dynamics. To investigate whether human partial-LOF mutants can be rescued to trigger apoptosis by increasing their expression level, we transfected p53-null cells with increasing amounts of wild-type and mutant p53-expressing GFP-adenoviruses. As a human cancer model, we chose the p53-deficient Saos-2 osteosarcoma cell line, in which the apoptosis-deficiency of cooperativity and other partial-LOF mutants has originally been described [44, 45]. To ensure that adenoviral p53 expression is close to physiological, we carefully titrated the adenoviruses to reach protein expression levels that are similar to activated endogenous wild-type p53. For this reference, we treated p53 wild-type HCT116 colorectal cancer cells with the highly-specific Mdm2-inhibitor Nutlin-3a (Supplementary Fig. S3A and B). To control for adenovirus-induced toxicity, the total adenovirus dose was kept constant by adding GFP-expressing control adenovirus and validating GFP-protein levels to be equal in all samples accordingly. We measured the cell-cycle inhibitory properties by EdU immunofluorescence staining for S-phase cells and, in parallel, used a real-time Annexin V-based split-nanoluciferase complementation assay to quantify apoptosis in a time-resolved manner. When expressed at equal levels, similar to activated endogenous p53 in HCT116 cells, all patient-derived cooperativity mutants (R181C, R181H, and R181L) as well as E180R (corresponding to murine E177R) were indistinguishable from wild-type p53 (WT) in causing cell-cycle arrest (Fig. 8A) but showed the expected decrease in apoptosis characteristic for partial-LOF mutants (Fig. 8B). By comparing with a titration of WT protein (Fig. 8C), peak apoptosis levels were decreased by at least 60% for R181L and by >80% for the other mutants. Importantly, for R181L and R181H apoptosis was fully rescued to WT-levels when mutant protein expression was raised 4- to 8-fold (Fig. 8D and E). For E180R and R181C, apoptosis was partially rescued at 8-fold higher expression, reaching 60% and 30% peak levels, respectively. Moreover, while all partial-LOF mutants were able to transactivate *CDKN1A/p21* above mock level, they were strongly compromised at inducing *BBC3/Puma* mRNA when expressed at the same level as WT (Fig. 8F). Notably, *BBC3/Puma* expression was fully or partially rescued to the WT-level at 8-fold higher expression (Fig. 8F).

Moreover, the rescue was confirmed at the level of promoter binding by chromatin immunoprecipitation (Fig. 8G). When expressed at WT-like levels (1× mut), all partial-LOF mutants yielded a binding signal significantly different from the IgG control at the *CDKN1A/p21* promoter, but not at the *BBC3/Puma* promoter. When expressed at 8× higher levels, p53 binding increased and reached at both promoters a level that was significantly different from the background and comparable to binding of wild-type expressed at 1×.

These experiments reveal that multiple patient-derived partial-LOF mutants can bind and transactivate a pro-apoptotic target gene promoter when expressed at increased levels, indicating that the previously reported transcriptional apoptosis-deficiency is not absolute and can be overcome by an increase in mutant protein level.

## Discussion

A pan-cancer *TP53* mutome analysis revealed a high prevalence of p53 partial LOF mutations that include p53 DNA binding cooperativity mutations affecting residues E180 and R181 (Fig. 1). RNA-seq profiling of the E180R (murine E177R) mutant demonstrated that its transcriptional activity is representative of the class of cooperativity mutants and validated this mouse strain as a suitable in vivo model for p53 partial-LOF mutations (Fig. 2). In tissues as different as pancreas, lung, and bone marrow the E177R mutant sensitized to *Ras* oncogene-driven tumorigenesis, highlighting the pathogenicity of a partial-LOF mutation. Interestingly, a similar degree of cancer susceptibility has also been observed in p53^neo^ mice which exhibit partial-LOF owing to reduced expression levels of wild-type p53 resulting from the intronic insertion of a neomycin cassette [15]. Levels of p53 expression in p53^neo/–^ and p53^neo/neo^ MEFs were 7.4% and 16.1% of the level of p53 expression in p53^+/+^ MEFs, resulting in a reduced median lifespan of 251 and 395 days, respectively [15]. As such, p53^neo^ mice are remarkably similar to p53^E177R^ mice, the resulting tumors are nevertheless notably different. Tumors in p53^neo^ mice were reported as negative for p53 by immunostaining, whereas tumors in p53^E177R^ mice were strongly immunopositive for p53 in all models we studied (Figs. 3–5). In this respect, E177R tumors were more similar to tumors with p53 hotspot mutations than tumors with reduced levels of wild-type p53.

Nevertheless, E177R tumors were very different from p53-null and hotspot-mutant tumors in their response to chemotherapy. While wild-type p53 can promote or diminish chemotherapy responses in a manner depending on cellular context and pathway activation [51], many hotspot-mutations exert GOF activities that render tumors more drug-resistant than the loss of p53 [7]. In contrast, E177R-mutant leukemias were more sensitive to standard chemotherapy than p53-null leukemias. The chemotherapy response was apoptotic and associated with induction of pro-apoptotic p53 target genes (Fig. 7C-G). This finding was rather surprising, given that E177R is unable to bind non-canonical REs in many pro-apoptotic target genes and E177R mice are characterized by a defect in p53-mediated apoptosis [30]. However, this apoptosis defect is not absolute and it was recently shown that in *Mdm2*-knockout embryos E177R triggers widespread apoptosis and leads to embryonic lethality [29]. Possible causes are the massive E177R stabilization caused by *Mdm2*-deficiency combined with the low apoptosis threshold of highly-proliferating embryonal tissues. We speculated that similar conditions are present in E177R leukemia cells. First, hematopoietic cells are exceptionally vulnerable to p53-mediated apoptosis as demonstrated by the strong hematopoietic phenotypes of mice with elevated p53 activity and the bone marrow toxicity of Mdm2 inhibitors [38, 39, 52, 53]. Second, E177R is constitutively stabilized in murine leukemia cells similar as cooperativity mutants in cancer patients. It can be assumed that sustained high-level expression of E177R restores binding to pro-apoptotic promoters by mass action, so that additional exogenous stress in the form of chemotherapy drives E177R leukemia cells into apoptosis. In support of this hypothesis, we experimentally validated that the apoptotic activity of E177R/E180R (and various patient-derived partial-LOF mutants) is rescued at elevated expression levels. Notably, non-transcriptional mitochondrial and cytoplasmic activities also contribute to p53-mediated apoptosis and support p53’s transcriptional apoptotic activity [54–56]. Furthermore, non-transcriptional apoptotic functions can be retained by tumorigenic p53 mutants and drive a beneficial chemotherapy response [32]. It is therefore conceivable that additional non-transcriptional functions of E177R further contribute to the survival-promoting therapy response seen in our AML mouse study.

In summary, our study further emphasizes the functional diversity among p53 mutations and reveals that partial-LOF mutations have a distinct translational impact on the course of tumorigenesis and cancer therapy and should be distinguished from classical hotspot mutations when using p53 mutations for predicting prognosis or deciding between treatment options.

## Materials and methods

### Animal experiments

All mouse experiments were performed according to the German Animal Welfare Law (TierSchG) and approved by the Regierungspräsidium Gießen based on recommendations of their animal welfare committee. The size of animal cohorts was determined a priori based on a biometric plan aimed at achieving statistically significant results at an effect size (Cohen’s d) of 1, error of α = 0.05, and power of 1-β = 0.80. The approved animal study protocols specified pre-established humane endpoint criteria. Animals that reached the endpoint before the end of the experiment were excluded from the analysis (or censored in survival studies). In treatment studies, animals were randomized to treatment cohorts by investigators and monitored by caretakers blinded to cohort allocation. Mice were housed in open cages, on a 12 h light/dark cycle, fed a standard housing/breeding diet (Altromin), and received water ad libitum.

Conditional 129 S2-*Trp53*^*tm1Thst*^/Thst (*Trp53*^*LSL-E177R*^) knock-in mice have been described [30]. Homozygous *Trp53*^*LSL-E177R/LSL-E177R*^ mice with the intact LSL cassette (deficient for p53 expression) were used as p53-null controls. For the PDAC model, triple-transgenic animals were generated through the breeding of double-heterozygous B6.129S/Sv-Kras^tm4Tyj^/JThst (*Kras*^*+/LSL-G12D*^), B6.FVB-Tg(Pdx1-cre)6Tuv/JThst (*Pdx-Cre*) with homozygous 129S2-*Trp53*^tm1.1Thst^/Thst (*Trp53*^*E177R/E177R*^) or B6.129P2-*Trp53*^tm1Brn^/JThst (*Trp53*^*flox/flox*^) animals. For the LUAD model, B6.129S2-*Kras*^tm2Tyj^/NciThst (*Kras*^*LA1*^) knock-in mice were intercrossed with *Trp53*^*E177R/E177R*^ or *Trp53*^*LSL-E177R/LSL-E177R*^ animals. MRI-assisted lung examination was done with a 7 T Clinscan 70/30 USR (Brucker) as described before [53]. Generation of the leukemia mouse model, monitoring of disease development by BLI, and therapy were performed as described earlier [32, 37]. Experiments with leukemia control cohorts (*Trp53*^+/+^ and *Trp53*^–/–^) depicted in Fig. 6 have been described previously [32] and were performed in parallel to the *Trp53*^*E177R/E177R*^ cohort. 129X1Sv/J and 129.B6F1 albino mice were used as recipients in the leukemia model.

### Real-time apoptosis assay

Saos-2 cells were infected in white 96-well clear-bottom plates with p53-expressing Ad-vectors and cultures in a CO_2_-independent medium (Thermo Fisher). RealTime-Glo™ Annexin V Apoptosis Assay (Promega) reagents were added 1 h post-infection according to the manufacturer’s protocol. Luminescence was recorded over 72 h after infection using a Cytation 3 Plate Reader (Biotek).

### Statistical analysis

GraphPad Prism 8 software was used for statistical analysis. Graphs show mean values obtained with n technical or biological replicates, and error bars in all figures represent standard deviation (SD), unless indicated otherwise. To assess comparisons between multiple groups, ANOVA followed by Dunnett’s multiple comparisons test was performed. To assess comparisons between two groups, the Student’s t-test was used. Data were validated to have similar variances and meet the assumption of (log)normal distribution by Kolmogorov-Smirnov test. p-values <0.05 were considered significant. Gene Set Enrichment Analyses were evaluated based on enrichment scores (ES) and FDRq-values, considering FDRq<0.25 as significant.

## Supplementary information

Supplementary Information File

Supplementary Table S1
