# Supplementary material for: p53 partial loss-of-function mutations sensitize to chemotherapy
Source: Oncogene. 2021 Dec 14;41(7):1011–23. doi: 10.1038/s41388-021-02141-5 (PMC8837531; doi:10.1038/s41388-021-02141-5)
Supplement: Supplementary file 1 — Supplementary Information File [file 41388_2021_2141_MOESM1_ESM.pdf]

SUPPLEMENTARY INFORMATION

p53 Partial Loss-of-Function Mutations Sensitize to Chemotherapy

Boris Klimovich[1,*], Nastasja Merle[1,*], Michelle Neumann[1], Sabrina Elmshäuser[1], Andrea Nist[2], Marco Mernberger[1], Daniel Kazdal[3], Albrecht Stenzinger[3], Oleg Timofeev[1], and Thorsten Stiewe[1,2]

[1] Institute of Molecular Oncology, Universities of Giessen and Marburg Lung Center (UGMLC), Member of the German Center for Lung Research (DZL), Philipps-University, Marburg, Germany;

[2] Genomics Core Facility, Philipps-University, Marburg, Germany;

[3] Institute of Pathology, Heidelberg University Hospital, Translational Lung Research Center Heidelberg (TLRC-H), Member of the German Center for Lung Research (DZL), Heidelberg, Germany.

[*] These authors contributed equally.

Correspondence to Oleg Timofeev: timofeev@staff.uni-marburg.de; Thorsten Stiewe: stiewe@uni-marburg.de.

SUPPLEMENTARY MATERIALS AND METHODS

Cell culture and gene transfer

For experiments, all cells were maintained at ambient oxygen in a humidified cell culture incubator (37°C, 5% CO_2) in high-glucose Dulbecco's Modified Eagle's Medium (DMEM) supplemented with 10% fetal bovine serum (FBS, Sigma-Aldrich), 100 U/ml penicillin and 100 µg/ml streptomycin (Life technologies). Recombinant adenoviral vectors co-expressing GFP and FLAG-tagged p53 mutants were produced in Ad293 cells (Agilent) using the pAdEasy system (Agilent) and used for infection of Saos-2 cells as described before [1]. Ad-vectors were titrated to precisely regulate the amount of ectopically expressed p53, while equalizing the total amount of Ad-vector encoding GFP only, using Western blotting and GFP flow cytometry.

EdU immunofluorescence analysis

Saos-2 cells were plated in black clear-bottom 96-well plates (Greiner), infected with Ad-vectors and harvested 30 hours post-infection. S phase cells were labeled with 10 µM EdU (Baseclick) added to the culture medium 1.5 hours before fixation. Cells were fixed at room temperature (RT) on plates (3.7% PFA in PBS, 15 min), washed twice (3% BSA in PBS) and permeabilized (0.5% Triton-X100, 20 minutes). EdU was detected using Click-It cocktail (4 mM $CuSO_4$, 1 µM Eterneon Red Azide, 100 mM ascorbic acid in PBS) for 30 minutes at RT. After washing twice with 3 % BSA in PBS, nuclei were counterstained with 0.1 µg/ml DAPI in PBS. Fluorescence images were obtained using the high-content automated BD Pathway (BD Biosciences) microscope. Images were taken as a 3x3 montage with A647 (EdU) and DAPI filters for each well and analyzed with the Attovision software (BD Biosciences) to determine the percentage of EdU-positive cells.

Western blot and immunohistochemistry (IHC)

Cells were collected 18 hours after infection and lysed in NP-40 Lysis Buffer (50 mM Tris-HCl, 150 mM NaCl, 5 mM EDTA, 2% NP-40, pH 8.0) supplemented with protease inhibitor (complete ULTRA tablets EASYpack, Roche) and sonicated with a Bioruptor (Diagenode) for 5 min. Western blotting was performed as described [2] using the following antibodies against: human p53 (DO-1, Santa Cruz Biotechnology [SC], 1:5000), GFP (B-2, #sc-9996, SC, 1:500), β-actin (AC-15, #ab6276, Abcam, 1:10000). Detection was performed with

secondary anti-mouse or anti-rabbit IgG-HRP (GE Healthcare, 1:5,000) and SuperSignal ECL kit (Thermo Fisher). β-actin was detected using goat anti-mouse Alexa-488 conjugate (A-11029, Thermo Fisher). Formalin fixed and paraffin embedded (FFPE) mouse samples were processed for histology and IHC as described [2] using the following antibodies against: cleaved caspase-3 (#9661, Cell Signaling, 1:100), mouse p53 (NCL-p53-505, Leica Microsystems, 1:1000), human p53 (DO-1, SC, 1:1000), BrdU (BU1/75(ICR1), #OBT0030G, 1:100), GFP (ab6556, Abcam, 1:500), p19Arf (5-C3-1, #sc-32748, SC 1:50), phosho-Erk (E-4, #sc-7383, SC, 1:100), biotinylated goat anti-rabbit IgG (E0 432, DAKO, 1:500) and biotinylated rabbit anti-mouse IgG (31834, Invitrogen, 1:500). Human specimen of pulmonary adenocarcinoma (ADC) used in this study were resected at the Thoraxklinik at Heidelberg University Hospital and diagnosed according to the criteria of the current WHO Classification (2015) for lung cancer [3] in the Institute of Pathology at Heidelberg University Hospital. The use of FFPE samples was approved by the ethics committee of Heidelberg University (S-145/2017). Immunohistochemical staining for p53 in human tumor specimen was carried out automatically using a BenchMark ULTRA autostainer (Ventana Medical Systems Inc., Tucson, AZ, USA) according to the manufacturer's instructions, in order to apply a ready to use solution of the anti-p53 mouse monoclonal antibody, clone Bp53-11 (F. Hoffmann-La Roche AG, Basel, CH) to the sections. IHC images were acquired with the Leica Aperio Versa slide-scanner and Leica Aperio eSlide Manager software v. 1.0.3.37. Analysis of IHC images was done using Aperio ImageScope software v. 12.3.2.8013. Quantification was performed using the Positive Pixel Count Algorithm v.9 and calculated as the ratio $N_{positive}/N_{total}$ pixels in 10 fields of view (1000X1000 pixel each) per sample.

mRNA expression analysis by RT-qPCR

RNA was isolated from cells or tissue samples using the RNeasy Mini kit (Qiagen). cDNA was generated with the SuperScript VILO cDNA Synthesis Kit (Invitrogen) and used for quantitative PCR with SYBR Green (Thermo Fisher Scientific) and the following primer pairs: mouse β-*Actin* 5'- CATTGCTGACAGGATGCAGAAGG (sense), 5'-TGCTGGAAGGTGGACAGTGAGG (antisense); *Cdkn1a/p21* 5'-CAAGAGGCCCAGTACTTCCT (sense), 5'-ACACCAGAGTGCAAGACAGC (antisense); *Bbc3/Puma* 5'- GTACGAGCGGCGGAGACAAG (sense), 5'-GCACCTAGTTGGGCTCCATTTCTG (antisense), human *GAPDH* 5'-CTATAAATTGAGCCCGCAGCC (sense), 5'- ACCAAATCCGTTGACTCCGA (antisense); *BBC3/Puma* 5'- ACCTCAACGCACAGTACGAG (sense), 5'-

GAGATTGTACAGGACCCTCCA (antisense). Gene expression was analyzed on a LightCycler 480 (Roche) with the ΔΔCt method using β-*Actin* or *GAPDH* for normalization.

Chromatin immunoprecipitation

Saos-2 cells growing on 15 cm dishes were fixed at 80% confluency with freshly prepared 18.5% (w/v) paraformaldehyde (PFA) for 10 min at RT, aiming at a final concentration of 0.88% (v/v) PFA for fixation. Crosslinking of DNA and proteins was terminated by quenching unreacted PFA by addition of glycine to 125 mM end concentration and further incubation of the cells for 5 min at RT. Cells were rinsed twice with ice-cold PBS, scraped off the dishes in 1 ml PBS supplemented with protease inhibitor. After pelleting at 700 x g for 5 min at 4°C, cells were lyzed at a concentration of $2x10^7$ cells/ml in SDS lysis buffer containing protease inhibitor. 250 µl lysate per tube was sonicated to shear DNA to a fragment size of 200 - 1000 bp, using the Sonicator Bioruptor Twin UCD-400 (Diagenode) for 5 cycles of 30 s ON/ 30 s OFF. After sonication, cell debris was pelleted by centrifugation for 10 min at 10,000 x g at 20°C. Supernatant containing the sheared chromatin was aliquoted á 100 µl and either frozen at - 80°C for later use or directly processed. For each antibody in the subsequent immunoprecipitation, one 100 µl aliquot was used and diluted 1: 10 with dilution buffer. Pre-clearing was performed for 1 h at 4 °C using 50 µl blocked Protein G-coupled sepharose beads (1:1 slurry with 20% EtOH) per sample. Supernatant of pelleted beads (3000 x g, 1 min, 4°C) was transferred into a new tube and 1% was removed as input DNA and stored at 4°C. Samples were rotated over night at 4°C with 2.5 µg antibody: human p53 (DO-1, Abcam ab 1101) or isotype control (E5Y6Q, Cell Signaling #61656). For precipitation, protein-DNA complexes were bound to blocked beads (50 µl per sample) for 4 h at 4°C on the next day. Hereafter, beads were washed once with each Low Salt, High Salt and LiCl Immune Complex washing buffers and twice with TE buffer. Crosslinking was reverted by incubating the immunoprecipitated samples and inputs at 99°C for 10 min with 100 µl of 10% (w/v) Chelex 100 solution (in ddH2O). Proteins were digested by proteinase K for 30 min at 55°C and this enzyme was subsequently inactivated at 99°C for 10 min. DNA was eluted in two steps with ddH2O, first with 55 µl and then with 100 µl each time by centrifugation (1 min at 12,000 x g). Analysis of bound DNA fragments was performed using qPCR with 1 µl DNA per reaction with the primer pairs: *BBC3/Puma* 5’-GCGAGACTGTGGCCTTGTGT (sense), 5’- CGTTCCAGGGTCCACAAAG (antisense); *CDKN1A/p21* 5’- AGCAGGCTGTGGCTCTGATT (sense), 5’-CAAAATAGCCACCAGCCTCTTCT (antisense).

RNA seq data analysis

RNA was isolated using the RNeasy Mini kit (Qiagen) and RNA quality was assessed using the Experion RNA StdSens Analysis Kit (Bio-Rad). RNA-seq libraries were prepared from total RNA using the QuantSeq 3′ mRNA-Seq Library Prep Kit FWD for Illumina (Lexogen) in combination with the UMI Second Strand Synthesis Module for QuantSeq FWD (Illumina, Read 1) (Lexogen) according to the manufacturer's instructions. Quality of sequencing libraries was controlled on a Bioanalyzer 2100 using the Agilent High Sensitivity DNA Kit (Agilent). Pooled sequencing libraries were quantified with digital PCR (QuantStudio 3D, Thermo Fisher), sequenced on the NextSeq550 platform (Illumina) with 75 base single reads and archived at EBI ArrayExpress (E-MTAB-10216, E-MTAB-10245).

Unique molecular identifiers (UMI) were extracted from the sequenced reads and the first four nucleotides corresponding to the QuantSeq FWD-UMI 3' spacer were removed. Trimmed reads were mapped to the *Homo sapiens* (revision 96, GRCh38) or *Mus musculus* (revision 92, mm10) Ensembl reference genome, using STAR (version 2.6.1d). After alignment, UMIs were deduplicated using UMI-tools (version 1.0.0), UMI per gene were quantified and normalized to CPM (counts per million). Genes that did not yield a CPM count of at least one in a single sample were discarded. Differential expression was assessed using DEseq2 (version 1.22.2) and obtained p-values were corrected via Benjamini-Hochberg correction. Genes with $\log2FC \geq 1$ as well as corrected p-values smaller than 0.05 were considered differentially expressed. For heatmaps, expression values were z-transformed and genes were clustered using k-means clustering. Clustering and dimensionality reduction was performed using the sklearn package (version 0.22.1). Gene set enrichment analysis was performed using Molecular Signatures Database (MSigDB) gene sets (Table S1) and GSEA software (version 3.0) [4, 5].

Supplementary References

1 Schlereth K, Beinoraviciute-Kellner R, Zeitlinger MK, Bretz AC, Sauer M, Charles JP *et al*. DNA binding cooperativity of p53 modulates the decision between cell-cycle arrest and apoptosis. *Mol Cell* 2010; 38: 356-368.

2 Timofeev O, Schlereth K, Wanzel M, Braun A, Nieswandt B, Pagenstecher A *et al*. p53 DNA binding cooperativity is essential for apoptosis and tumor suppression in vivo. *Cell Rep* 2013; 3: 1512-1525.

3 Travis WD, Brambilla E, Nicholson AG, Yatabe Y, Austin JHM, Beasley MB *et al*. The 2015 World Health Organization Classification of Lung Tumors: Impact of Genetic, Clinical and Radiologic Advances Since the 2004 Classification. *J Thorac Oncol* 2015; 10: 1243-1260.

4 Liberzon A, Subramanian A, Pinchback R, Thorvaldsdottir H, Tamayo P, Mesirov JP. Molecular signatures database (MSigDB) 3.0. *Bioinformatics* 2011; 27: 1739-1740.

5 Liberzon A, Birger C, Thorvaldsdottir H, Ghandi M, Mesirov JP, Tamayo P. The Molecular Signatures Database (MSigDB) hallmark gene set collection. *Cell Syst* 2015; 1: 417-425.

6 Kato S, Han SY, Liu W, Otsuka K, Shibata H, Kanamaru R *et al*. Understanding the function-structure and function-mutation relationships of p53 tumor suppressor protein by high-resolution missense mutation analysis. *Proc Natl Acad Sci U S A* 2003; 100: 8424-8429.

7 Kotler E, Shani O, Goldfeld G, Lotan-Pompan M, Tarcic O, Gershoni A *et al*. A Systematic p53 Mutation Library Links Differential Functional Impact to Cancer Mutation Pattern and Evolutionary Conservation. *Mol Cell* 2018; 71: 178-190.e178.

SUPPLEMENTARY FIGURES

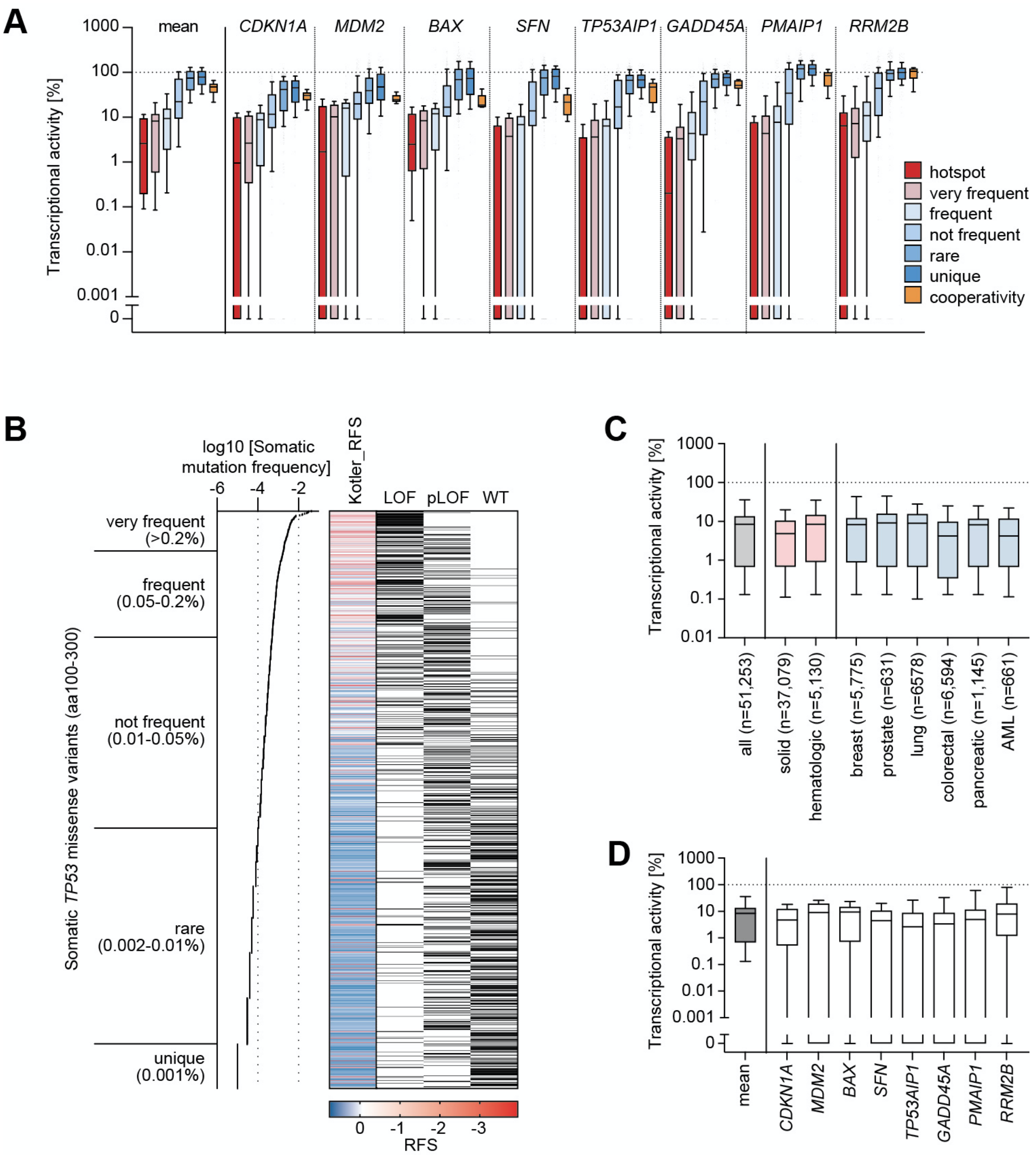

Supplementary Figure S1. Transcriptional activity of 1,209 missense mutations from the UMD p53 mutation database. A, Transcriptional activity of somatic *TP53* mutations grouped according to mutation frequency in cancer patients. **B**, Relative fitness score (RFS) of *TP53* mutations ordered from top to bottom by mutation frequency in cancer patients. LOF, loss of function: RFS>0; pLOF, partial loss of function: 0<RFS<-2; WT, wildtype-like: RFS<-1. **C-D**, Transcriptional p53 activity in cancer patients with *TP53* missense mutations according to **C** tumor type and **D** target gene. Transcriptional activity data from [6], relative fitness scores from [7].

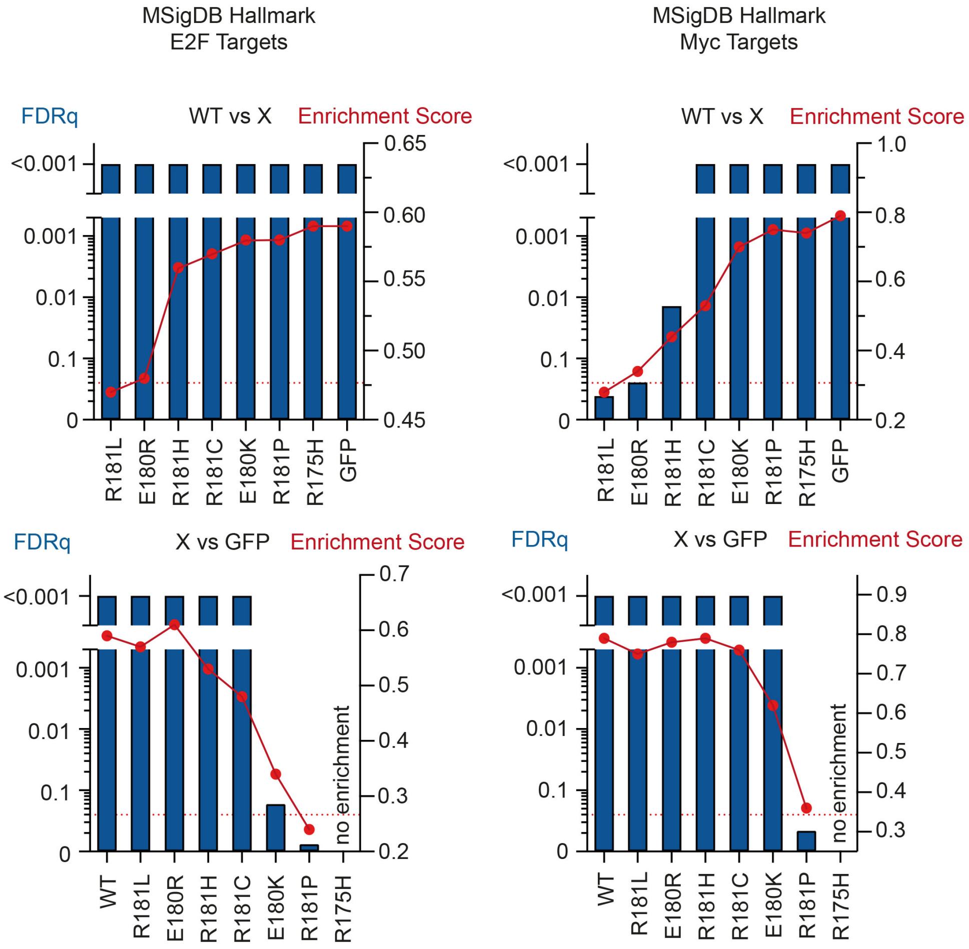

Supplementary Figure S2. Partial loss of transcriptional repression in p53 cooperativity mutants. RNAseq data from Fig. 2A were used for gene set enrichment analysis (GSEA). Depicted are the false discovery rate (FDRq, blue bars) and enrichment score (red curve) for the MSigDB Hallmark E2F and Myc Target gene sets in pairwise comparisons between cells expressing wild-type p53 and different mutants (upper panels) or all variants versus GFP-transfected (p53-null) cells (lower panels).

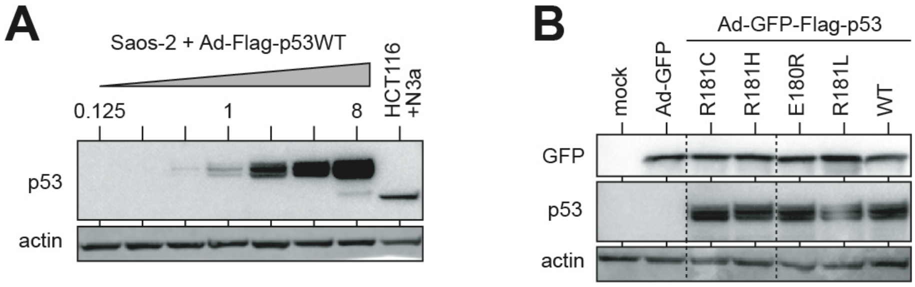

Supplementary Figure S3. Elevated protein levels rescue the transcriptional apoptosis defect of partial-LOF mutants. **A**, Western blot of FLAG-tagged wild-type p53 ectopically expressed in Saos-2 cells upon vector titration (0.125-8X). Protein lysate from p53 wild-type HCT116 cells treated with Nutlin 3a was included as a reference for endogenous p53 expression levels. **B**, WB confirming equal vector load (GFP) and p53 protein levels for all p53 variants ectopically expressed in Saos-2 cells (4X titer is shown).
